# A Controlled Trial of Polyglytone 6211 versus Poliglecaprone 25 for Use in Intradermal Suturing in Dogs

**DOI:** 10.3390/ani11113094

**Published:** 2021-10-29

**Authors:** Pagona Gouletsou, Nikitas Prassinos, Lysimachos Papazoglou, Polychronis Kostoulas, Apostolos Galatos

**Affiliations:** 1 Clinic of Obstetrics and Reproduction, Faculty of Veterinary Science, School of Health Sciences, University of Thessaly, Trikalon 224, 43100 Karditsa, Greece; 2 Companion Animal Clinic, School of Veterinary Medicine, Faculty of Health Sciences, Aristotle University of Thessaloniki, Thessaloniki, S Boutyra 11, 54627 Thessaloniki, Greece; ngreen@vet.auth.gr (N.P.); makdvm@vet.auth.gr (L.P.); 3 Laboratory of Epidemiology, Faculty of Public Health, School of Health Sciences, University of Thessaly, Terma Mavromichali, 43131 Karditsa, Greece; pkost@uth.gr; 4 Clinic of Surgery, Faculty of Veterinary Science, School of Health Sciences, University of Thessaly, Trikalon 224, 43100 Karditsa, Greece; agalatos@vet.uth.gr

**Keywords:** canine, polyglytone 6211, poliglecaprone 25, intradermal, wound healing

## Abstract

**Simple Summary:**

The choice of suture material for skin closure can affect the final cosmetic outcome, the risk of wound infection, and other complications in companion animals. We assessed two commercially available suture materials, namely Caprosyn and Monocryl, for use in suturing the skin of dogs, by using cosmetic, clinical, and histological evaluation. The results indicate only minimal differences between the two products, although better scores were achieved after using Monocryl. Both were found sufficient for use in intradermal suturing in dogs. The earlier absorption of Caprosyn, compared to Monocryl, did not have any additional beneficial effect on wound healing and scar appearance in dogs.

**Abstract:**

The objective of this work was the comparative evaluation of the healing process after employing 4/0 poliglecaprone 25 and 4/0 polyglytone 6211 in a continuous intradermal suture pattern. Ten Beagle dogs were used, in which skin incisions were created surgically and subsequently were sutured by means of continuous intradermal pattern using polyglytone 6211 or poliglecaprone 25 suture. Cosmetic, clinical, and histologic scores were evaluated. The cosmetic appearance of the wounds was blindly evaluated on days 7, 14, 28, 180, 360, 730, and 1095. On the same days, tissue biopsy was performed for histological evaluation. Clinical evaluation was performed initially daily, then weekly, monthly, and finally yearly, till day 1095. The clinical appearance of the intradermal pattern with both sutures was initially very good, deteriorated in the second post-operative month and thereafter improved. The cosmetic, clinical, and histological differences between the two suture materials were minimal and statistically insignificant. Polyglytone 6211 is sufficient for use in intradermal suturing in dogs. However, its earlier absorption compared to poliglecaprone 25 did not have any beneficial effect on cutaneous wound healing and scar appearance in the experimental animals.

## 1. Introduction

Suturing for the primary closure of wounds intends to approximate the edges of tissues, aiming to form a functional and cosmetically acceptable wound scar. Recent studies have highlighted the importance of continuous intradermal suturing pattern for cosmetically effective skin closure [1,2,3,4,5]. However, in a study performed over time, which evaluated healing of wounds sutured with intradermal suture pattern [1], it has been observed that appearance of wounds could deteriorate subsequently to the first month post-operatively, possibly due to the irritation of the skin caused by the suture material [1]. The choice of suture material for skin closure can potentially affect the final cosmetic outcome, the risk of wound infection, and other complications. There is currently insufficient evidence regarding the optimal suture material for intradermal suturing. Furthermore, controlled evaluations comparing continuous intradermal suturing, performed with various suture materials, are limited [3,5,6,7,8,9,10,11]. Moreover, among these, in only a few the suturing technique was assessed by applying objective clinical criteria or histological evaluation [1,9,10,12,13]. The impact of a rapidly absorbable suture used during intradermal suturing has been studied in humans [14,15,16] and rats [17]; nevertheless, no studies are available highlighting the potential impact of rapidly absorbable sutures on the cosmetic outcomes of intradermal suturing in dogs. Further, polyglytone 6211 has not been tested in the dog and no data exist on the impact of its implantation to this species.

The objective of this work was the comparative evaluation of the healing process after employing 4/0 poliglecaprone 25 and 4/0 polyglytone 6211 in a continuous intradermal suture pattern. Our hypothesis was that use of polyglytone 6211 would be superior to poliglecaprone 25 for intradermal suturing, because, as it is absorbed earlier (56 days versus 100 days post-operatively), it would probably cause less skin irritation at the wound area.

## 2. Materials and Methods

### 2.1. Animals and Experimental Design

Ten healthy Beagles (5 males and 5 females), aged 1–5 years, were used in this work. During the first month of the study, each dog was individually housed; subsequently, they were transferred to pens with 3–4 animals. Strict measures of pain management were taken and their handling and use during the experiments and the housing of them for all the required time period was according to the laws of EU.

At the beginning of the study, all dogs were clinically healthy. A detailed dermatological examination did not reveal relevant lesions; no wounds or scars were evident in the areas of the animal body, where it was planned to perform the experimental incisions. A routine hematological testing was performed; this included a full blood test (CBC) and full blood biochemical examination, i.e., profile for liver function (alkaline phosphatase (ALP), alanine transferase (ALT), aspartate aminotransferase (AST), *γ*-glutamyl transferase (GGT) and total bilirubin (Tbili)), kidney function (blood urea nitrogen (BUN), creatinine and phosphorus), glucose and blood serum proteins and electrolytes (K, Na, Cl). Additionally, a urine test was performed. No abnormal findings were seen in laboratory examinations and all results were within reference values for all animals. No animal in the study received any medication for 30 days prior to the start of the work. Clinical and laboratory examinations as detailed above were thereafter performed every month or every quarter, respectively, throughout the study.

For all surgical procedures, relevant analgesia and anesthesia were performed (medetomidine (dose rate: 20 μg kg^−1^, IM, (intramuscularly); Domitor, Pfizer Hellas, Athens, Greece), morphine (dose rate: 0.5 mg kg^−1^, IM; Morphine hydrochloride, DEMO Hellas, Athens, Greece), thiopental (dose rate: 5–7 mg kg^−1^, IV (intravenously); Pentothal, Abbott Hellas, Athens, Greece), isoflurane (1.5–2%; Aerrane, Baxter, Compton, United Kingdom) in oxygen (1 L min^−1^)).

All surgical incisions in all animals were performed by the principal author (P.G.), who also made the sutures. The skin and the subcutaneous tissues were incised (12 cm long) on the lateral surface of each hind limb of the experimental animals, parallel to the axis of the femur. One incision was sutured with continuous intradermal pattern, using 4/0 poliglecaprone 25 (P25); for the other, continuous intradermal suture pattern with 4/0 polyglytone 6211 (P6211) was used. Each of the above two combinations had been sealed into an envelope; before surgery an envelope was picked and the relevant combination was performed.

The continuous intradermal suture pattern, which involved a mattress, continuous and horizontal, within the dermal layer with burying of knots, was performed in a standard procedure: the first knot of suture material was buried within the subcutaneous tissue 4 mm from the wound commissure; suture material was then directed towards the incision start in the middle of the dermis. The needle was positioned horizontally, through the upper dermis, by taking special care to confirm that no material of the suture crossed the epidermis. During the final suture, the needle was pointed backwards; an Aberdeen-type knot was employed to fix the suture at the subcutaneous tissue. Then, the tip of the suturing material was pointed towards the subcutaneous tissue at the lateral side of the incision and outside the skin. Therefore, the knot was displaced from the healing layer of the skin, whilst the suture was cut, leveled with the skin surface.

Immediately post-surgery and up to the 14th post-operative day, Elizabethan collars were placed on the dogs, with the aim to preventing licking of wounds. For analgesia, morphine was administered (0.5 mg kg^−1^, IM, 6 times daily on day 1 and 4 times on day 2). Antibiotics were not given to any animal.

### 2.2. Cosmetic Evaluation

Cosmetic appearance of the sutured wounds was assessed by two other authors (N.P., L.P.), who were blinded to the precise procedures performed at each of them. Photographs of the wounds, taken 7, 14, 28, 180, 360, 730, and 1095 days post-operatively, were assessed; note that the final assessment was performed in 5 of the 10 dogs. The assessment and evaluation were based on a one (1) to five (5) visual comparative scoring system (1 given for the best scar, 5 given for the worst scar among all animals, as seen on each evaluation point). Lack of hair regrowth was also studied on a zero (0) to three (3) scoring system (0: regrowth, 1: a little lack of regrowth, 2: mild lack of regrowth, 3: severe lack of regrowth). Scores allocated by each of the two assessors were summed to obtain the ‘total cosmetic appearance score’ for each wound on each evaluation day. As the scores were given on a comparative scale, a higher total score indicated a poorer cosmetic appearance of the wound (Figure S1).

### 2.3. Clinical Evaluation

Clinical evaluation of the wounds was performed post-operatively, every day for 14 days and then 18, 21, 25, 28, 35, 42, 49, 60, 90, 120, 150, 180, 240, 365, 730, and 1095 days after surgery (i.e., for 3 years). Erythema, skin thickening, abscessation or inflammation, presence of exudate, scar width, comedones, hyperpigmentation, wound dehiscence, and lack of hair regrowth were evaluated during clinical evaluation. The scoring system detailed in Table S1 was employed. The clinical examination of the wounds was always performed by the principal author (P.G.). In a case that any of the above were present unevenly on an incision, at least two measurements were made; in such cases, the mean value of the measurements was taken.

### 2.4. Histological Evaluation of the Healing Process

First, 7, 14, 28, 180, 365, 730, and 1095 days after surgery [1,6,18,19], tissue samples [20] were taken from the incisions by means of punch biopsy. In total, 7 samples were obtained from each wound, as detailed herein. The first biopsy was performed 1 cm further to the upper commissure of the incision; subsequent biopsies were made distally, 1 cm apart. After collection, the tissue samples were bisected, under magnification, in a perpendicular plane to the incision. The tissue samples were then prepared for histological examination by using standard procedures [1,5,9].

During the histological examination, the following parameters were evaluated: necrosis, edema, epithelial gap, presence of suture, inflammation, presence of fibroblasts, tissue reaction around the suture, scar width, epithelial thickness, collagen synthesis, and angiogenesis. A scoring system presented in Table S2, was used.

### 2.5. Data Management and Analysis

In order to detect any potential differences between the two suturing materials, the experimental period was divided into four time-periods: period A, from day 1 to 28 after surgery (i.e., when inflammation, debridement, proliferation, and repair took place), period Β, from day 32 to 60 after surgery (i.e., when the early maturation stage took place), C, from day 90 to 300 after surgery (i.e., when the median maturation stage was present), and D, from day 365 to 1095 after surgery (i.e., when the late maturation stage was established).

Scores for cosmetic, clinical, and histological results were added to produce a cumulative score, representative of each time-period. For the overall cosmetic score for each time period, the scores given by each of the two assessors were summed. Then, the scores for exudate, comedones, inflammation/abscessation, lack of hair regrowth, and pigmentation given during the clinical examination were added to produce a clinical score for each time period. Further, skin thickening, erythema, scar width, and dehiscence were transformed to a scale from 0 to 3, corresponding to the 25th, the 50th, and the 75th, respectively, percentile of the distribution of total values. These were subsequently summed up to the representative score [1].

For histological assessment, the scores given for presence of edema, inflammatory reaction, tissue reaction around the suture material, thickness of the epidermis at the area of wound healing, epidermal gap, and scar width were summed. Values for the thickness of the epidermis at the area of wound healing, the epidermal gap and the scar width, before summation, were again transformed to a scale from 0 to 3. Tissue necrosis was not included in the scoring scheme, as there was no sign of it in any sample, and the lesion was omitted from further analysis. Collagen deposition, fibroblast presence, and angiogenesis also were not included into the summation, as they were not found to differ between the two suturing schemes [1].

For the comparison of the suturing techniques throughout the duration of the study, the total score of each scheme was assessed on days 7, 14, 28, 180, and 365 post-surgery, by summing up, on each occasion, the total scores for the cosmetic, the clinical, and the histologic evaluation [1].

Observations are clustered at three levels: repeated measurements performed throughout the time (level 1) at the same animal (level 2) for the two different sutures (level 3). A non-parametric approach was used, as measurements were not distributed normally. Specifically, the Friedman test (non-parametric ANOVA [21]) was employed to evaluate if the median values of each parameter differed between the suturing techniques used for each time period of the study. The threshold for statistical significance was set at *p* < 0.05. All analyses were performed in a Stata version 8.2 software (Stata Corporation, College Station, TX, United States of America).

## 3. Results

### 3.1. Cosmetic Evaluation

No significant difference was evident regarding the cosmetic evaluation between the two suturing materials during any period of the study (*p* > 0.2018 for all comparisons). Note that there was no difference between the scores returned by each of the two assessors, at any stage of the study (*p* > 0.08 for all comparisons). Details are in Table 1.

### 3.2. Clinical Evaluation

#### 3.2.1. Skin Thickness

**Figure 1:**
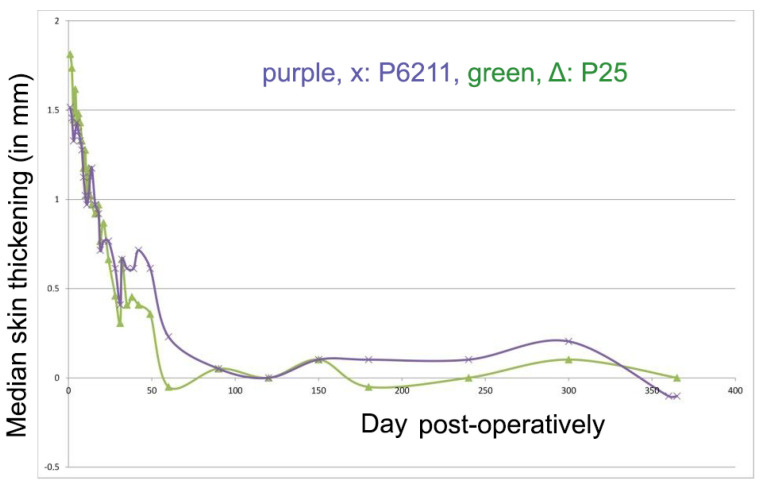
Median skin thickening (mm) for each suturing material (purple: P6211; green: P25) during the study.

Skin thickness after use of each suture material is shown in Figure 1. No significant difference was observed at any time period.

#### 3.2.2. Erythema

**Figure 2:**
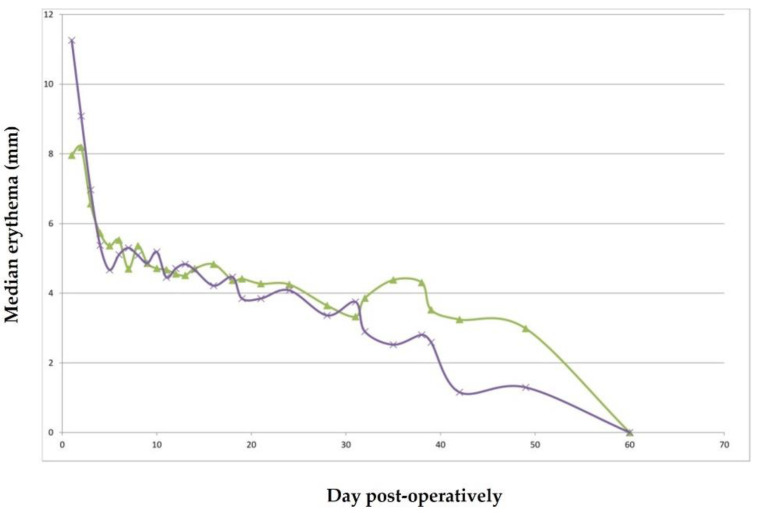
Median erythema (mm) for each suturing material (purple: P6211; green: P25) during the study.

Erythema was observed in all incisions from day 1 to 50 post-operatively. Width of the erythema was more extensive for P6211 during the first 2 days post-operatively, a trend that was reversed during the second post-operative month (Figure 2). No significant difference was observed at any time period.

#### 3.2.3. Width of the Scars

**Figure 3:**
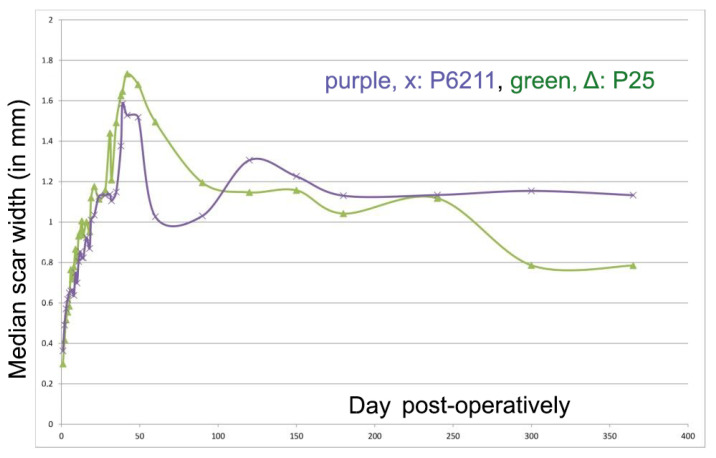
Median scar width (mm) for each suturing material (purple: P6211; green: P25) during the study.

The width of the scars progressively increased until day 50 and thereafter gradually decreased. However, with P6211, it increased again after day 90 and stayed larger than that of P25 afterwards (Figure 3). These differences, however, were not statistically significant.

#### 3.2.4. Inflammation and Abscessation

Inflammation was observed in a small number of animals, in which incisions were sutured with both materials. No significant differences were seen between the two materials used.

#### 3.2.5. Exudate

Exudate was observed in all incisions with inflammation and microabscessation. The exudate appeared to be serosanguineous to purulent. No significant differences were found between the two materials.

#### 3.2.6. Comedones

Comedones were seen in 6 of the 10 animals used in the study. In these, comedones were evident in both incisions. In both incisions, comedones were observed from day 14 until day 180, however a larger number of them was observed the second post-operative month. No significant differences were seen between the materials.

#### 3.2.7. Hyperpigmentation at Wound Area

Hyperpigmentation was observed in all the wounds, with the exception of two incisions sutured with P25, and usually occurred from the second month to one year post-operatively. The intensity of pigmentation differed from mild to moderate. No significant difference was detected between the materials.

#### 3.2.8. Hypotrichosis

Hypotrichosis at the incision line was seen with both suture materials, usually from day 21 until 60 (less often, from 10 to 180); hypotrichosis was mild or moderate. No significant difference was seen between the two suture materials.

#### 3.2.9. Cumulative Results of the Clinical Evaluation

Detailed results for the scores assigned during the clinical evaluation of the wounds, for each of the two suturing materials and throughout the study are presented in Table 2. The median score and the interquartile range of the total clinical evaluation for each material during each study period are also shown in Table 2. No significant differences were observed in any time period between the two materials employed.

### 3.3. Histological Evaluation

#### 3.3.1. Necrosis

No necrosis was observed in any tissue sample evaluated.

#### 3.3.2. Epithelial Gap

Epithelial gap was observed in 4 tissue samples obtained on day 7 from the P25 (median: 0.3 mm, max: 1.3 mm) and in 5 samples obtained from P6211 (median: 0.6 mm, max: 1.8 mm). However, no significant differences were seen between the two suture materials.

#### 3.3.3. Edema

Edema was observed in 13/20 samples from both materials obtained on day 7, and in only one sample sutured with P25 on day 14; the difference was not significant.

#### 3.3.4. Inflammation

Inflammation was evident in all the samples collected on day 7. Then, it was also seen in 14/20 of samples collected 14 days and in 9/20 of samples collected obtained 28 days post-surgery. Again, no significant difference was seen between the two materials.

#### 3.3.5. Presence of Suture Material and Tissue Reaction

Suture material was evident in 42/60 samples from the wounds. The suture material or its implantation site was seen in samples taken up to day 28 post-surgery. During period A, the P25 material was associated with milder tissue reaction around the suture than the P6211 material (1 (1–2) and 1.5 (1.5–2), respectively). However, this difference was not found to be significant.

#### 3.3.6. Epithelial Thickness

On day 7, epithelial thickness was × 3.5 the normal with P25 and × 3 with P6211. On day 14 was × 2 and × 2.25, and on day 28 was × 1.5 and × 1.75, respectively. No significant difference was detected in period A between the two materials.

#### 3.3.7. Scar Width

In most of the samples taken on days 7 and 14 post-surgery, and in 50% of samples taken on day 28, it was not possible to determine the width of scar tissue, as the result of the wound’s infiltration by inflammatory cells and fibroblasts. No significant differences were seen between the two suture materials at any time period.

#### 3.3.8. Collagen Deposition, Fibroblast Presence, Angiogenesis

No significant differences were evident in the collagen deposition score, score for the presence of fibroblasts, and score for angiogenesis between the two suture materials at any time period.

#### 3.3.9. Cumulative Results of the Histological Evaluation

Detailed results for the scores assigned during the histological evaluation of the wounds for each of the two suturing materials and throughout the study are presented in Table 3. The scores (median values and interquartile ranges) of total score for histological evaluation for each suture material in each time period are also presented in Table 3.

### 3.4. Cumulative Evaluation

**Figure 4:**
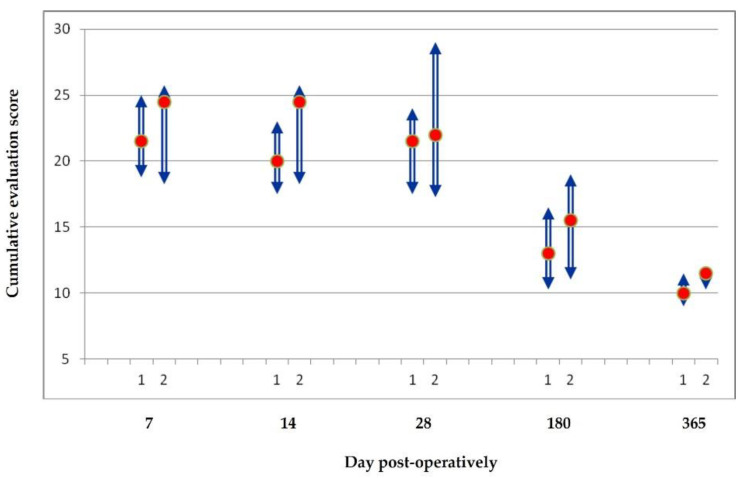
Cumulative evaluation score (median score (red dot) and interquartile range (column)) for each suturing material (1: P25, 2: P6211) during the study.

The scores (median values and interquartile ranges) for the cumulative evaluation for each suture material on each biopsy day are presented in Figure 4. Throughout the study, there was a tendency for the total evaluation scores to be better for wounds sutured with P25 than that for wounds sutured with P6211, but this difference was not significant (*p* > 0.075).

## 4. Discussion

Various suturing techniques are available for use and the specific advantages of each of them for wound closure have been described in relevant papers. In general, intradermal suture pattern is considered to have superior cosmetic results, principally because the epidermis of the sutured skin is not penetrated [22], whilst local inflammation is small; moreover, a good approximation of wound edges can be achieved, which results in development of a small scar [23]. Nevertheless, there is a lack of objective studies of the impact of suture material on the intradermal suturing [1].

The initial reaction of stitched tissue is a reflection of the amount of injury, inflicted by passage of the needle and suture [24]. Assuming the same technique, tissue, and other reactive factors, e.g., absence of infection, the reaction would be the same for all sutures during the first five to seven days, if not longer. The subsequent reactions, however, refer to the medium- to long-standing response of tissues to sutures [24,25].

Presence of a foreign body, for example the suture, within the tissues can result in inflammatory reaction, which can affect the stage of proliferation and repair, possibly resulting in reduced wound strength and creation of a hypertrophic scar. Healing of a tissue initially involves a stage of inflammation, which lasts up to a week after the injury, as the wound then enters the stage of proliferation and healing. However, when there is suture material in the wound, the inflammatory reaction persists as long as it remains in the tissue. To a large extent, the severity of inflammation depends upon the properties of the suture [25,26]. In addition, the presence of a suture in the tissue could increase the chance of infection by acting as a “pathway” for microorganisms to penetrate the tissue and by creating a mechanical barrier to the movement of inflammatory cells to the site of infection. Therefore, one may think that sutures with smaller absorption time, as P6211 in the present study, may inflict less tissue inflammation, i.e., of lesser severity and shorter duration. However, in the present study, there were no statistically significant differences in the inflammatory response between P6211 and P25.

Poliglecapron 25, which results from co-polymerization of glycolic acid and ε-caprolactone, is a synthetic single-strand absorbable suture. It has excellent characteristics when handled, i.e., it is pliable, has no “memory”, and allows the formation of stable knots [27], while causing little tissue reaction; this is characterized by the presence of macrophages and fibroblasts mainly [28]. It is also inactive and does not show capillary action, while it has a low coefficient of friction [27]. At first, the suture resistance to tension is high, but it decreases quickly (by 75% within 14 days), to be completely lost 21 days after implantation in the tissues, while its complete absorption takes place in 91–119 days after application [27,28,29].

Polyglytone 6211, which consists of glycolic acid, ε-caprolactone, trimethylene carbonate, and lactide, is a synthetic single-stranded absorbable suture. It is easy to handle and allows the formation of stable knots, while causing a mild tissue reaction. These characteristics make it ideal for suturing the skin, especially for intradermal suturing [30]. This suture material has a high resistance to tension during implantation, which is satisfactory until the 10th day and is lost by the 21st day post-operatively. The fact that it is completely hydrolyzed by day 56 also makes it one of the fastest absorbing sutures [30].

Polyglytone 6211 had not been studied in dogs, and the present study was the first to evaluate its use in this species. The investigation of the effects of its use in dogs is important, as differences between species in the reaction to suture material are common. Furthermore, it was important to compare it with a gold standard for intradermal skin suturing that had been previously tested in dogs, as is poliglecapron 25 [1]. The present study showed that polyglytone 6211 is a suture material suitable for use in intradermal skin suturing in dogs and that wound healing after its use has no significant differences in comparison to poliglecapron 25.

The formation of scar tissue at the wound site is a normal development of healing but degrades the aesthetic effect of healing. In many cases, in which there is tension at the edges of the wound, there is an enlargement of the thin scar that initially forms [31]. It seems that the application of an intradermal suture reduces the size of the scar, as it can support the edges of the wound for a longer period of time and can achieve better contact between them [4]. In the present study, the scar width was slightly larger at the incisions sutured with P25 compared to those sutured with P6211 for the first two months post-operatively; whilst after the first year, the scar width was slightly larger at the incisions sutured with P6211 compared to those sutured with P25, although all differences were statistically non-significant. The wider scar width with P6211 after one year may be the result of the shorter support of the wound area due to faster absorption. Polyglytone 6211 is completely hydrolyzed by day 56 also and is one of the fastest absorbed sutures [30].

A few authors studied these two suture materials for use in intradermal suture pattern in other species. In rats, Van Heerden [17] tested histologically the inflammatory response in 10 animals on days 2 and 10 after intradermal suturing with P6211 or P25. The presence of neutrophils, macrophages, and lymphocytes was evaluated around the suture and given a grade from 1 to 4. It was found that there were no differences between the sutures on the control days, nor were there any significant differences between the days for the same suture, although on the 2nd day neutrophils predominated, while on the 10th day so did macrophages. In two cases of P25 use, mast cells and eosinophils were observed, which the author assumed were the result of an allergic reaction. No such findings were observed in our histological evaluation, as only fibroblasts, neutrophils, and macrophages were detected around the sutures. Furthermore, Van Heerden [17] monitored the incisions for a small time period, so limited information is given.

Obermair et al. [15] compared the aesthetic effect and post-operative complications in human patients who had staples and, furthermore, had undergone continuous intradermal suturing with either P25 or P6211. The incisions were scored by the patients and the surgeon 1 and 6 weeks and 3 months post-operatively, on a scale from 0 to 100. Patients evaluated worse the incisions sutured with P6211 compared to those sutured with P25 until the 6th postoperative week, however, they found no difference 3 months after surgery. The evaluation of the surgeon was slightly better at all times for P25 compared to P6211, but without a statistically significant difference. In addition, the likelihood of postoperative complications did not differ between techniques. Their findings in humans are similar with ours in dogs.

Naghshineh et al. [14] evaluated, for up to 12 weeks, the clinical and cosmetic outcome in human patients after skin suturing with separate intradermal sutures with P6211 and P25. The evaluation was performed by the patients themselves and blindly by two clinical experts, and showed no differences in aesthetic appearance, scar size, and complications, such as infection, rupture, granulation, necrosis, serous collection, and hematoma. The only difference observed was the more frequent expulsion of the suture from the scar at the incisions sutured with P25 compared to those sutured with P6211. In contrast, in the present study, neither expulsion of the suture from the scar, nor any other complications, as the aforementioned, were noticed. The same authors also found that after the 2nd post-operative month the intradermal suture with P25 suture had a consistently better clinical evaluation than with P6211, albeit the difference not being statistically significant; these findings are in full accordance with ours in dogs.

To our knowledge, no research has been performed to compare the two suture materials in the dog. The present study aimed to provide relevant information, as, given that the reaction to suture materials and suture techniques differs between species, it is important for surgeons to have access to fully relevant information.

In the present study, the cosmetic evaluation did not differ significantly between the two suture materials. Hence, the results were not supportive of the hypothesis that a rapidly absorbed suture would benefit cosmesis of the healing area.

During the clinical assessment, skin thickening at the incision line was seen with both suturing materials, starting on day 1 after surgery. Thickening was more intense during the 1st month and decreased subsequently to the 3rd postoperative month, with no difference evident between the two suture materials. It was noticed that thickening of the skin increased again in the second post-operative month with both materials, probably due to the presence of the sutures inside the healing area that caused irritation. Afterwards, even as P6211 had been absorbed, the thickening of the wound area sutured with P6211 was larger than that of P25, showing that early absorption did not provide any benefit with regard to that aspect.

Erythema at the incision line was observed from day 1 to 60 and did not differ between the two suture materials. This finding during the first post-operative days was probably a result of impairment of the superficial vascular plexus of the dermis, which resulted in turn in extravasation of blood cell and erythema. Erythema occurs usually during wound healing; however, in this case, it might have lasted longer consequently to irritation caused by the implanted suture material. Sylvestre et al. [5] have reported that the intradermal suture pattern can induce, in dogs, an erythema more intense than the simple interrupted suture pattern immediately after surgery, which then declines on days 10–14 post-surgery.

Immediately post-surgery, the incision site was covered by a scab as the wound contracted. Later, after the sloughing off of that scab, an increase in width was seen in the scar, independently of suture material and until day 40, thereafter gradually decreasing. The early absorption of P6211 caused a thinner scar between days 60 and 90; however, afterwards scar widening was observed, probably due to the early loss of strength and flattening of the wound area. Scar width was stabilized after day 300 with both suture materials. Therefore, in the long term, early absorption of the suture material did not have a beneficial effect on scar width. Breed et al. [32], who compared Vicryl Rapid (absorption in 42 days) with P25, also found that rapidly absorbing suture materials give significantly wider scars.

Comedones were also found during the study, and were evident from day 14 to 180, and more frequently and in higher numbers during the 2nd month post-operatively. Smeak [4] has indicated that trauma and irritation of the *stratum basale* of the epidermis, during suturing can induce cyst formation in there. Gouletsou et al. [1], who also found comedones when suturing with 3/0 and 4/0 P25, reported that formation of comedones was possibly secondary to a rupture of hair follicles caused by the needle or the suture or as the result of inflammatory reaction. In the present study, comedones were observed mainly in the 2nd postoperative month, when both sutures were intact, so early absorption of P6211 did not reduce their presence.

Hyperpigmentation at the incision line was seen in all wounds during the study, between days 28 and 180 after surgery. The initial finding related to pigment deposition at the wound area occurred 7 to 14 days after surgery, with the maximum concentration of melanocytes occurring a few months later [33,34]. In the present study, hyperpigmentation was probably induced by cytokines, which are normally produced during wound healing, and can stimulate melanin production [35,36], while afterwards, wound color returned to normal.

Total clinical evaluation, which summarized the various components of the clinical findings, indicated that until day 60, suturing with P6211 gave a smaller score than P25, whilst afterwards the opposite occurred.

Histological evaluation of the wound site did not reveal any differences between the two suture materials. Epithelial gap was observed in four samples from P25 and in five samples from P6211, on day 7, and full epithelialization afterwards. Gouletsou et al. [1] also reported similar findings; in contrast, Pope [37] reported the finding of full epithelialization 48 h after surgery and Kirpensteijn et al. [3] within 7 days after skin suturing. Tissue reaction around the suture was less intense for P25 than for P6211 during the 1st month; however, the difference was not statistically significant. The scores of histological findings did not differ between the two suture materials at any time period. Van Winkle et al. [7] and Gouletsou et al. [1] also did not report differences in collagen production between wounds closed with different sutures. Total histological evaluation also showed no differences between the two sutures.

Finally, in order to compare these suture materials over time, the total representative score of each technique was evaluated on 7, 14, 28, 180, and 365 days after surgery, by adding on each occasion the cosmetic, clinical, and histological total scores obtained on each observation day. This evaluation has the limitation of including several different parameters that are connected to each other; but, on the other hand, it provides a broad scale for evaluation of the effects. The total evaluation score in each biopsy day was smaller for the wounds sutured with P25 than for those sutured with P6211, without, however, any statistically significant difference.

## 5. Conclusions

In conclusion, polyglytone 6211 was found to be a safe and effective suture material for use with intradermal suture pattern in dogs. However, its earlier absorbance than poliglecaprone 25 suture was not associated with an effect on wound healing and scar appearance. Both suture materials are sufficient for use in intradermal suture technique in dogs.

## Figures and Tables

**Table 1:** Cosmetic scores for each suturing material, given by two different assessors and total cosmetic evaluation throughout the study (median values and interquartile length (in brackets)).

Parameter	Post-Operative Period					
	1–28 Day		90–300 Day		365–1095 Day	
	Suturing Material					
	P25	P6211	P25	P6211	P25	P6211
1st assessor						
Cosmetic	3.0 (3–4)	3.0 (3–4)	2.0 (2–3)	2.0 (2–3)	2.0 (2–2)	2.0 (2–2)
Hypotrichosis	1.5 (1–2)	1.3 (1–2)	1.0 (0–1)	1.0 (0–1)	0.0 (0–1)	0.0 (0–1)
2nd assessor						
Cosmetic	3.0 (3–3)	3.0 (3–4)	3.0 (2–3)	3.0 (2–3)	2.0 (2–2)	2.0 (1–2)
Hypotrichosis	1.0 (1–2)	1.3 (1–2)	10. (0–2)	1.0 (1–1)	0.3 (0–1)	0.3 (0–1)
Total cosmetic evaluation result	8.0 (8–10)	9.0 (7–11)	7.0 (4–9)	7.8 (6–8)	4.5 (4–6)	4.8 (4–6)

**Table 2:** Scores of clinical evaluation for each suturing material throughout the study (median values and interquartile length (in brackets)).

Parameter	Post-Operative Period							
	1–28 Day		32–60 Day		90–300 Day		365–1095 Day	
	Suturing Material							
	P25	P6211	P25	P6211	P25	P6211	P25	P6211
Skin thickening (mm)	1.3 (0.9–1.5)	1.1 (0.9–1.2)	0.4 (0.0–0.9)	0.5 (0.2–0.8)	0.1 (−0.4–0.2)	0.1 (0.0–0.4)	0.0 (−0.3–0.1)	0.0 (0–0)
Erythema (mm)	4.5 (4.3–5.3)	4.6 (4.1–5.3)	3.1 (0.0–4.4)	1.7 (0.0–3.9)	0.0 (0–0)	0.0 (0–0)	0.0 (0–0)	0.0 (0–0)
Scar width (mm)	0.9 (0.6–1.0)	0.7 (0.6–0.8)	1.5 (1.4–1.8)	1.4 (1.0–2.0)	1.1 (0.9–1.6)	1.1 (0.5–1.6)	0.8 (0.6–1.0)	1.1 (0.5–1.4)
Dehiscence (cm)	0.0 (0–0)	0.0 (0–0)	0.0 (0–0)	0.0 (0–0)	0.0 (0–0)	0.0 (0–0)	0.0 (0–0)	0.0 (0–0)
Exudation (score 0–3)	0.0 (0–0)	0.0 (0–0)	0.0 (0–0)	0.0 (0–0)	0.0 (0–0)	0.0 (0–0)	0.0 (0–0)	0.0 (0–0)
Abscessation (score 0–3)	0.0 (0–0)	0.0 (0–0)	0.0 (0–0)	0.0 (0–0)	0.0 (0–0)	0.0 (0–0)	0.0 (0–0)	0.0 (0–0)
Comedones (score 0–3)	0.0 (0–0)	0.0 (0–0)	0.5 (0–2)	0.5 (0–2)	0.0 (0–0)	0.0 (0–0)	0.0 (0–0)	0.0 (0–0)
Hypotrichosis (score 0–3)	0.0 (0–0)	0.0 (0–0)	1.3 (1–2)	1.8 (1–2)	0.0 (0–0)	0.0 (0–0)	0.0 (0–0)	0.0 (0–0)
Hyperpigmentation (score 0–3)	0.0 (0–0)	0.0 (0–0)	0.0 (0–0)	0 (0–0.5)	1 (0–1.5)	1 (0–1.5)	0.0 (0–0)	0.0 (0–0)
Total clinical evaluation result	5.5 (5–6)	4.5 (4–6)	7.3 (6–10)	5.8 (4–9)	3.0 (2–5)	3.5 (2–5)	1.0 (1–2)	2.7 (1–3)

**Table 3:** Results of histological evaluation for each suturing material throughout the study (median values and interquartile length (in brackets)).

Parameter	Post-Operative Period					
	1–28 Day		90–300 Day		365–1095 Day	
	Suturing Material					
	P25	P6211	P25	P6211	P25	P6211
Edema (mm)	0.0 (0–0)	0.0 (0–0)	0.0 (0–0)	0.0 (0–0)	0.0 (0–0)	0.0 (0–0)
Inflammation (score 0–3)	1.0 (0–1)	1.0 (1–1)	0.0 (0–0)	0.0 (0–0)	0.0 (0–0)	0.0 (0–0)
Collagen production (score 0–3)	2.0 (2–2)	2.0 (2–2)	3.0 (3–3)	3.0 (3–3)	3.0 (3–3)	3.0 (3–3)
Presence of fibroblasts (score 0–3)	3.0 (3–3)	3.0 (3–3)	2.0 (2–3)	2.0 (2–3)	2.0 (2–2)	2.0 (2–2)
Angiogenesis (score 0–3)	2.0 (1–2)	2.0 (2–2)	1.0 (1–1)	1.0 (1–1)	1.0 (1–1)	1.0 (1–1)
Tissue reaction around suture (score 0–3)	1.0 (1–2)	1.5 (1.5–2)	0.0 (0–0)	0.0 (0–0)		
Presence of suture (score 0–3)	3.0 (3–3)	3.0 (3–3)	0.0 (0–0)	0.0 (0–0)		
Epidermal thickness (×normal)	2.0 (1.5–2.5)	2.0 (2–2.5)	1.2 (1–1.5)	1.0 (1–1.2)	1 (1–1.2)	1 (1–1)
Epithelial gap (mm)	0 (0–0)	0 (0–0)				
Scar width (mm)	0.7 (0.7–0.9)	0.9 (0.8–1.3)	0.7 (0.7–0.9)	0.8 (0.7–1.8)	0. 7 (0.6–0.9)	0.6 (0.5–0.9)
Total histological evaluation result	6.5 (5–7)	7.0 (6–7)	4.0 (4–4)	4.5 (3–7)	4.0 (3–4)	3.0 (3–4)

## Supplementary Materials

The following are available online at https://www.mdpi.com/article/10.3390/ani11113094/s1, Figure S1: Photographs from the wounds. Table S1: Scoring system of clinical examination. Table S2: Scoring system of histological examination.
