# Supplementary material for: A Controlled Trial of Polyglytone 6211 versus Poliglecaprone 25 for Use in Intradermal Suturing in Dogs
Source: Animals (Basel). 2021 Oct 29;11(11):3094. doi: 10.3390/ani11113094 (PMC8614295; doi:10.3390/ani11113094)
Supplement: Supplementary file 1 [file animals-11-03094-s001.zip › animals-1422199-supplementary.pdf]

A Controlled Trial of Polyglytone 6211 versus Poliglecaprone 25e for Use in Intradermal Suturing in Dogs

Pagona Gouletsou, Nikitas Prassinos, Lysimachos Papazoglou, Polychronis Kostoulas and Apostolos Galatos

Figure S1. Photographs from the wounds

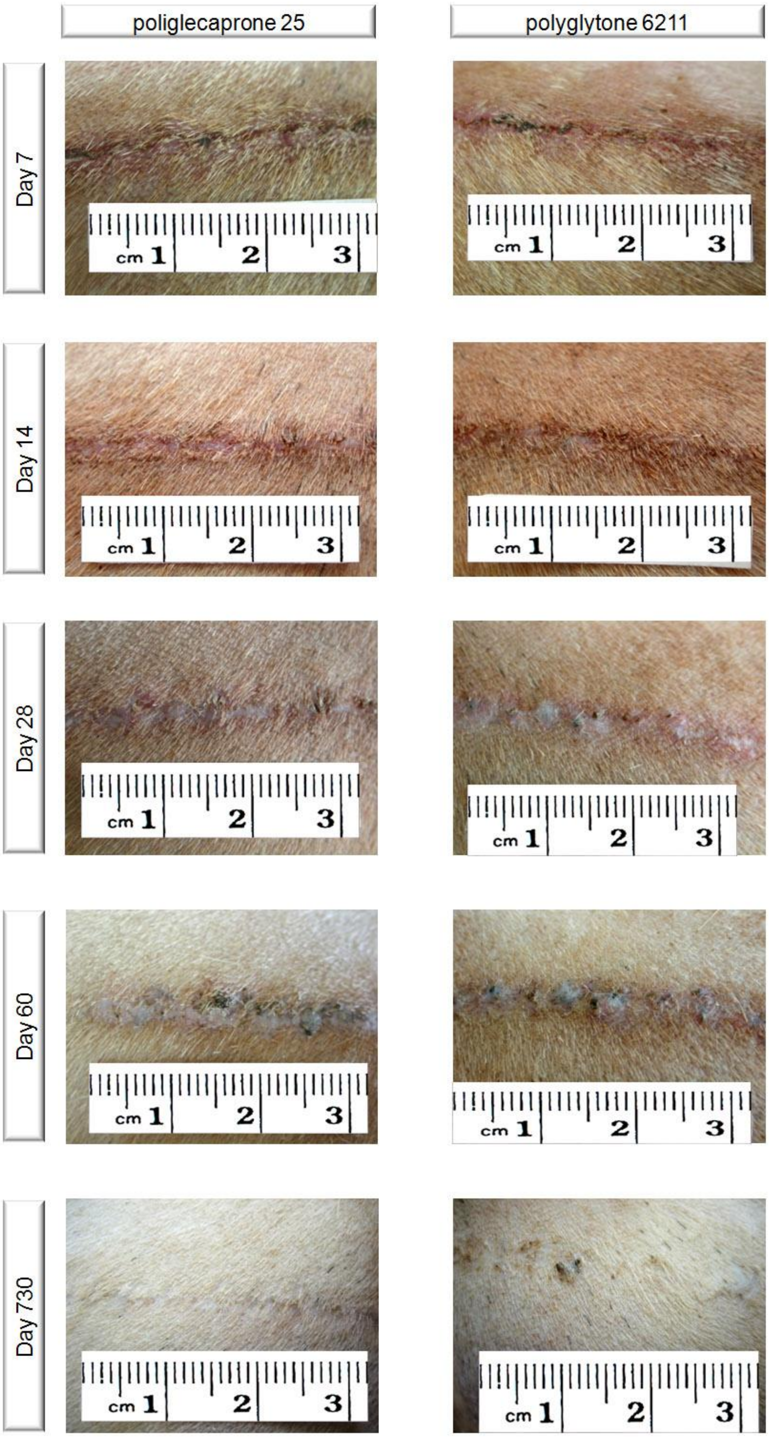

Table S1. Scoring system of the clinical examination

Parameter	Score 0	Score 1	Score 2	Score 3
Skin thickening	measured with a skin caliper at the central part of the incision (mm)			
Erythema	width of skin redness at the central part of the incision, measured with an electronic caliper (mm)			
Scar width	measured with an electronic caliper, in the central part of the incision, in mm			
Abscessation or inflammation	absence	mild inflammation	1-2 microabscesses	intense inflammation, or > 2 microabscesses
Exudate	absence	serosanguineous	seropurulent	purulent
Comedones	absence	1-3	4-6	> 6
Hyperpigmentation of the wound area, compared with the adjacent skin color	absence	mild	moderate	intense
Lack of hair regrowth	none	light	mild	severe
Wound dehiscence	Length (cm)			

Table S2. Scoring system of the histological examination

Parameter	Score 0	Score 1	Score 2	Score 3
Necrosis [1]	none identified	few scattered areas	multiple focal dense areas	necrosis present throughout the slide
Epithelial gap [2]	(mm)			
Oedema [4]	absence	slight separation of cells and collagen from each other in the wound tissue by non-stained or poorly stained acellular material	separation of approximately 30 to 50 μm by this acellular material	separation of > 50 μm
Inflammation [3]	<3 cells/field	3-10 cells/field	11-30 cells/field	>30 cells/field
	evaluated by scoring neutrophils, eosinophils, macrophages, lymphocytes, plasma cells, and mast cells detected in 10 high power fields (HPF) (400 ×)			
Tissue reaction around the suture [3]	0-2 cell layers around suture tract	3-5 cell layers around suture tract	6-10 cell layers around suture tract	>10 cell layers around suture tract
Epithelial thickness [3]	as a per cent proportion of normal epithelial thickness			
Scar width	mm			
Collagen synthesis [1,4]	no collagen	scant collagen bundles slightly separating fibroblasts	dense accumulations of collagen between fibroblasts	extensive separation of fibroblasts by collagen
Presence of fibroblasts [1,4]	<3 fibroblasts / field 400 ×	3-10 fibroblasts / field 400 ×	11-30 fibroblasts / field 400 ×	> 30 fibroblasts / field 400 ×
Angiogenesis [1]	<3 capillary buds / field 400 ×	3-10 capillary buds / field 400 ×	11-30 capillary buds / field 400 ×	>30 capillary buds / field 400×

1. Winkler, J.T.; Swaim, S.F.; Sartin, E.A.; Henderson, R.A.; Welch, J.A. The effect of a porcine-derived small intestinal submucosa product on wounds with exposed bone in dogs. *Vet Surg* **2002**, 31, 541-551, doi:10.1053/jvet.2002.34669.
2. Gouletsou, P.G.; Prassinos, N.N.; Papazoglou, L.G.; Kostoulas, P.; Galatos, A.D. Comparison of continuous intradermal with simple interrupted suture pattern: an experimental study in dogs. *Top Companion Anim Med* **2020**, 41, 100454, doi:10.1016/j.tcam.2020.100454.
3. Kirpensteijn, J.; Maarschalkerweerd, R.J.; Koeman, J.P.; Kooistra, H.S.; van Sluijs, F.J. Comparison of two suture materials for intradermal skin closure in dogs. *Vet Q* **1997**, 19, 20-22, doi:10.1080/01652176.1997.9694732.
4. Gillette, R.L.; Swaim, S.F.; Sartin, E.A.; Bradley, D.M.; Coolman, S.L. Effects of a bioactive glass on healing of closed skin wounds in dogs. *Am J Vet Res* **2001**, *62*, 1149-1153, doi:10.2460/ajvr.2001.62.1149.
